# Altered Lipid Composition of Surfactant and Lung Tissue in Murine Experimental Malaria-Associated Acute Respiratory Distress Syndrome

**DOI:** 10.1371/journal.pone.0143195

**Published:** 2015-12-01

**Authors:** Diletta Scaccabarozzi, Katrien Deroost, Natacha Lays, Fausta Omodeo Salè, Philippe E. Van den Steen, Donatella Taramelli

**Affiliations:** 1 Dipartimento di Scienze Farmacologiche e Biomolecolari, Università degli Studi di Milano, Milan, Italy; 2 Rega Institute for Medical Research, KU Leuven - University of Leuven, Leuven, Belgium; Instituto de Ciências Biomédicas / Universidade de São Paulo - USP, BRAZIL

## Abstract

Malaria-associated acute lung injury (MA-ALI) and its more severe form malaria-associated acute respiratory distress syndrome (MA-ARDS) are common, often fatal complications of severe malaria infections. However, little is known about their pathogenesis. In this study, biochemical alterations of the lipid composition of the lungs were investigated as possible contributing factors to the severity of murine MA-ALI/ARDS. C57BL/6J mice were infected with *Plasmodium berghei* NK65 to induce lethal MA-ARDS, or with *Plasmodium chabaudi* AS, a parasite strain that does not induce lung pathology. The lipid profile of the lung tissue from mice infected with *Plasmodium berghei* NK65 developing MA-ALI/ARDS, but not that from mice without lung pathology or controls, was characterized by high levels of phospholipids -mainly phosphatidylcholine- and esterified cholesterol. The high levels of polyunsaturated fatty acids and the linoleic/oleic fatty acid ratio of the latter reflect the fatty acid composition of plasma cholesterol esters. In spite of the increased total polyunsaturated fatty acid pool, which augments the relative oxidability of the lung membranes, and the presence of hemozoin, a known pro-oxidant, no excess oxidative stress was detected in the lungs of *Plasmodium berghei* NK65 infected mice. The bronchoalveolar lavage (BAL) fluid of *Plasmodium berghei* NK65 infected mice was characterized by high levels of plasma proteins. The phospholipid profile of BAL large and small aggregate fractions was also different from uninfected controls, with a significant increase in the amounts of sphingomyelin and lysophosphatidylcholine and the decrease in phosphatidylglycerol. Both the increase of proteins and lysophosphatidylcholine are known to decrease the intrinsic surface activity of surfactant. Together, these data indicate that an altered lipid composition of lung tissue and BAL fluid, partially ascribed to oedema and lipoprotein infiltration, is a characteristic feature of murine MA-ALI/ARDS and possibly contribute to lung dysfunction.

## Introduction

According to the WHO classification, deep breathing, respiratory distress and pulmonary oedema are among the clinical features occurring in severe malaria accompanied by lung complications [1–3]. Malaria-associated acute lung injury (MA-ALI), and its more severe form, malaria-associated acute respiratory distress syndrome (MA-ARDS) are prevalent in malaria-endemic areas with low transmission where adults get severe complications because protective clinical immunity is lacking [4, 5]. The precise incidence is not known, but it has been estimated between <2% to >25% in severe malaria cases, and mortality may be as high as 80% when mechanical ventilation is not available [5]. Up to 60% of severe zoonotic malaria cases caused by *P*. *knowlesi* develop MA-ARDS [6]. Knowledge about the pathogenesis of MA-ALI/ARDS is still limited, and especially the biochemical alterations associated with lung dysfunction have not been investigated, yet. Therefore, murine models have been developed, which are useful to perform detailed experiments to unravel the pathogenesis of MA-ALI/MA-ARDS [7, 8]. Although the histopathology and also the ultrastructure of murine MA-ARDS is similar to post-mortem analyses of human MA-ARDS cases, [9] the findings from mouse models must be confirmed in patient studies, since important differences may exist between human malaria and corresponding mouse models [10]. Inflammation and increased endothelial permeability are important features of both human and mouse MA-ALI/ARDS [3, 5, 8, 11–14]. High numbers of inflammatory cells are observed in lung biopsies from patients and mice that succumbed from this complication [5] and a significantly altered expression profile of inflammatory mediators was found in the lungs of mice with MA-ARDS [7]. Accumulation of hemozoin (Hz), the major waste product of hemoglobin degradation, in the lungs appears to be an important inflammatory stimulus contributing to MA-ARDS. Pulmonary Hz levels are significantly correlated with inflammation, increased lung weight and alveolar oedema in mice [15], and increasing amounts of Hz are observed on lung autopsies from African children with increasing disease severity [13]. Activated inflammatory cells and Hz can also cause oxidative stress, which may augment inflammation and contribute to vascular leakage and alveolar oedema [14–15]. Oxidative degradation of lipids results in the accumulation of reactive aldehydes, such as malondialdehyde (MDA) and 4-hydroxynonenal (4-HNE), which are highly cytotoxic [16, 17]. An altered lipid profile and increased levels of lipoperoxidation end products have been found in plasma from patients with ARDS of different aetiologies, however, no data are available on MA-ARDS [18, 19].

ARDS is also often associated with lung surfactant disorders, which can be observed soon after the initial injurious event and lead to increased surface tension, alveolar collapse and loss of liquid balance in the lungs [20, 21]. Pulmonary surfactant is synthesized by alveolar type II cells and consists of a lipoprotein complex with an essential role in reducing the surface tension at the air-liquid interface of lung epithelia and in lung immune defence. The lipid part is mainly composed of phospholipids (PLs), predominantly dipalmitoylphosphatidylcholine (DPPC) [22, 23] and high levels of phosphatidylglycerol (PG) whereas approximately 10% of the surfactant consists of specific proteins which contribute to the first-line defence against pulmonary pathogens to prevent infection and inflammation and aid in the adsorption of lipids. The newly synthesized surfactant is stored as tightly packed membranes known as lamellar bodies which are secreted into the alveolar hypophase as large multilamellar vesicles, known as the large aggregate (LA) surfactant fraction [23, 24]. The LA fraction acts as a reservoir for the surfactant layers that are spread as a surface film over the alveolar liquid-air interface. During breathing, LA is converted into small vesicles that are degraded by macrophages or recycled by the type II pneumocytes. These small vesicles are less surface active and constitute the small aggregate (SA) surfactant fraction [23].

Inflammation enhances the conversion of LA into SA [25, 26] and results in phospholipase A2 (PLA_2_) enzymatic degradation of mainly DPPC, the most abundant surfactant phospholipid, thereby generating equimolar amounts of lysophosphatidylcholine (LPC) and free palmitic acid [27, 28]. LPC, together with plasma proteins and specific free fatty acids that leak into the alveoli when vascular permeability is increased and the epithelial membrane is damaged, can interfere with normal surfactant activity and compromise lung compliance.

To deepen our understanding on the complexity of the pathological changes occurring during MA-ALI/ARDS, we explored the biochemical modifications and lipid alterations of lung tissue and the molecular organization and lipid composition of alveolar surfactant in C57BL/6J mice infected with *Plasmodium berghei* NK65 (*Pb*NK65), a well-established murine model of MA-ARDS. As a control, mice infected with *Plasmodium chabaudi* AS *(Pc*AS), a *Plasmodium* strain that does not induce lung pathology, were used [7].

## Materials and Methods

### Reagents

Unless differently specified all chemicals were purchased from Sigma (Milan, Italy). Standard fatty acid methylesters were purchased from Alltech (Milan, Italy) and silica gel plates (Kieselgel 60) for High Performance Thin layer Chromatography (HPTLC) from Merck (Darmstadt, Germany).

### Animals

#### Infection of mice with rodent malaria parasites

C57BL/6J mice (8–9 weeks of age, approximately 50% males and 50% females, all groups were age and sex-matched in all experiments) were infected by intraperitoneal injection of 10^4^ *Pb*NK65 or *Pc*AS infected red blood cells (RBCs). Peripheral parasitemia was determined by 10% Giemsa staining of blood smears followed by microscopic counting and expressed as percentage of infected red blood cells. Mice were sacrificed at day 8 or 10 post infection and blood was removed by heart puncture. All experiments were approved by the Animal Ethics Committee from the KU Leuven (License LA1210186, Belgium). All efforts were made to minimize suffering. Mice were euthanized by intraperitoneal injection of a lethal dose of sodium pentobarbital (200 μl at 60 mg/ml Nembutal).

#### Treatment of infected mice with dexamethasone (DEX)

Mice were treated with dexamethasone before the onset of MA-ARDS, according to previously published findings [7]. Briefly, dexamethasone sodium phosphate (DEX; CERTA, Braine-l’Alleud, Belgium) was dissolved in phosphate-buffered saline (PBS) and injected intraperitoneally at 80 mg/kg, in a volume of 200 μl daily, starting at day 6–7 post infection. As a control, another group of *Pb*NK65 infected mice was treated with PBS alone.

### Preparation of biological specimens

At the indicated time points, mice were sacrified. Blood was drawn by cardiac puncture into heparin-coated syringes and centrifuged to separate plasma and RBCs. Subsequently, left lungs were pinched off with a clamb. The bronchoalveolar lavage (BAL) and the perfusion with 0.15 M NaCl containing 0.2 mM butylhydroxytoluene (BHT) as an antioxidant, were performed only on the right lungs. The left lungs were weighted with a laboratory balance, whereas the right lungs were removed and homogenized in Precellys tubes in 6 volumes of a solution containing 20 mM Tricine, 250 mM sucrose, 5 mM EDTA, 0.2 mM BHT (pH 7.4) and a protease inhibitor cocktail (Sigma). Lung homogenates were further centrifuged at 1,000xg for 10 min (4°C), washed three times in the same buffer and the pooled supernatants were centrifuged at 100,000xg for 1 hour (4°C). The pellets, representing the enriched membrane fraction, were resuspended in 0.2 mM BHT and stored at -80°C until further analyses.

BAL fluid was collected from the right lungs by intra-tracheal instillation of an isotonic NaCl solution (3 x 0.6 ml) through a trachea cannula and centrifuged at 150xg for 10 min at 4°C to pellet the cells. The total (cell-free) supernatant was collected and immediately centrifuged at 12,000xg for 30 min (4°C) to obtain a Large Aggregate fraction (LA, pellet) and a Small Aggregate fraction (SA, supernatant) as described previously [29]. LA and SA fractions were lyophilized, resuspended in distilled water and stored at -80°C until further processing.

### Hemozoin determination

Perfused lungs were used for the determination of the Hz content by heme-enhanced chemoluminescence according to Deroost et al [30]. The Hz concentration (nM) in the homogenates was calculated from the calibration curve of the hematin concentration (nM) versus luminescence (events/sec) and subsequently expressed as pmol Hz/mg tissue.

### Lipid analyses

Lipids in plasma, BAL fluid and lung samples were extracted in organic solvents according to Folch [31]. The lipid extracts were dried under nitrogen, dissolved in chloroform and saved at -80°C until further analyses. Phospholipid (PL) phosphorus was determined by the Bartlett procedure [32] and PLs were separated by HPTLC plates with chloroform/methanol/acetic acid/water = 60:40:4:2 (v/v/v/v) as developing solvent. Spots were visualized with anisaldehyde [33] and quantified by densitometric analysis (Camag Reprostar 3). Cholesterol (Cho), triglycerides (TG) and cholesterol esters (ChoE) were quantified by densitometric analysis after separation by HPTLC in hexane/diethyl ether/acetic acid (90:10:1 by vol.) and visualized with a solution of 10% CuSO_4_ in 8% H_3_PO_4_. The fatty acid composition of the lipid fractions were analyzed by gas liquid chromatography (GLC) according to Corsetto et al. [34]. Lipid peroxidation was measured in the sample homogenates by determining the levels of thiobarbituric acid reactive substances (TBARS) as previously reported [35] and expressed as pmoles of malondialdehyde (MDA)/mg protein.

The collagen content of lungs, a marker of fibrotic degeneration, was determined by measuring hydroxyproline in the tissue homogenates [36].

### Statistical analysis

Results are reported as mean ± standard deviation (SD). Comparison between groups and replicates was performed using ANOVA followed by Tukey's multiple comparison test with GraphPad Prism 6.0 (Graphpad Software Inc., La Jolla, CA, USA). The differences were considered significant when p < 0.05.

## Results

### Disease course in mice infected with *Pb*NK65 or *Pc*AS

**Fig 1:**
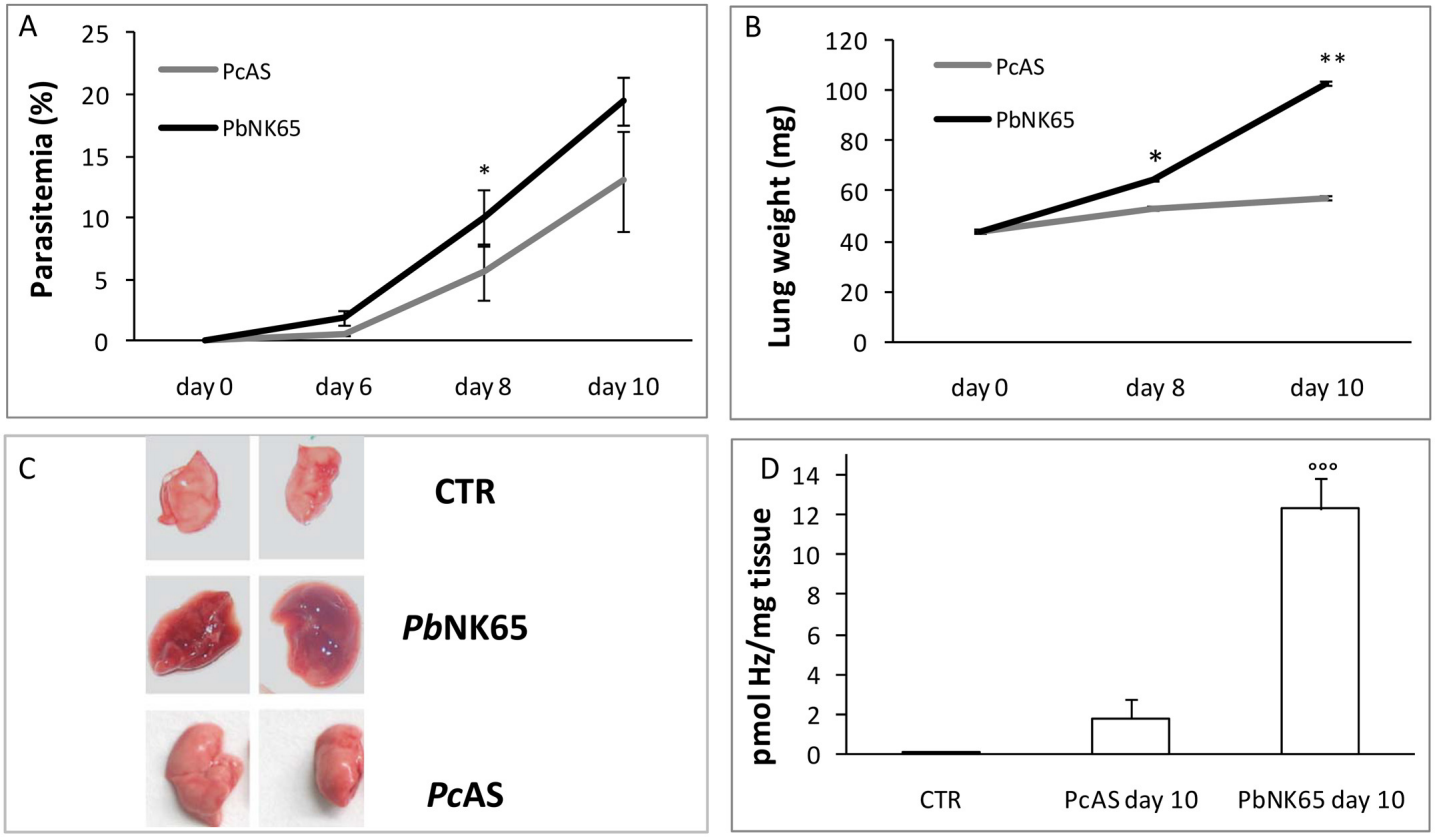
Disease course in mice infected with *Pb*NK65 or *Pc*AS. C57BL/6J mice were injected intraperitoneally with 10^4^ erythrocytes infected with *Plasmodium berghei (Pb*NK65) or *P*. *chabaudi* AS (*Pc*AS). Peripheral parasitemia (panel A) and weights of the left lungs (panel B) were determined at day 6, 8 and 10 post infection (n = 6–8 mice for each time point and strain). (panel C) Representative pictures of right lungs (not perfused) from uninfected (CTR) or infected mice (day 10 post infection). (panel D) Hz content in lung tissue (pmole Hz /mg lung tissue) at day 10 post infection. *p<0.05; **p<0.01; *** p<0.0001 vs CTR; °°°p< 0.01 *Pb*NK65 vs *Pc*AS.(n = 6–8 mice for each time point and strain).

C57BL/6J mice were infected intraperitoneally with 10^4^ RBC from mice infected with *Pb*NK65 or *Pc*AS and the development of parasitemia was monitored over time. The parasitemia increased steadily in *Pb*NK65-infected mice from day 0 to day 10, whereas a significantly lower parasitemia was detected in *Pc*AS-infected mice (Fig 1A). Corresponding to previous observations [7], extensive and lethal pulmonary pathology developed from day 8 post infection onwards in *Pb*NK65-infected mice as indicated by the increased weight and the swollen appearance of the lungs that was absent in *Pc*AS-infected mice (Fig 1B and 1C). *Pb*NK65 infection also resulted in a higher deposition of Hz in the lungs compared to *Pc*AS infected mice (Fig 1D). This contributes, in addition to haemorrhages, to the dark brown aspect of the lungs from *Pb*NK65-infected mice (Fig 1C) and is consistent with earlier observations [7].

### Alterations in the lipid profile of lung membranes from mice with MA-ARDS

To investigate whether malaria infection alters the lipid composition of the lung tissue, we infected mice with *Pb*NK65, which causes MA-ARDS, or *Pc*AS, which does not cause MA-ARDS. Furthermore, to investigate whether the lipid and FA changes observed in the lungs are specific for MA-ARDS or merely due to the parasite infection, one group of *Pb*NK65-infected mice was treated daily with 80 mg/kg DEX starting on day 7 after infection before the appearance of the pulmonary symptoms. This treatment regimen has been shown previously to prevent the development of pulmonary pathology without decreasing parasitemia [7].

**Fig 2:**
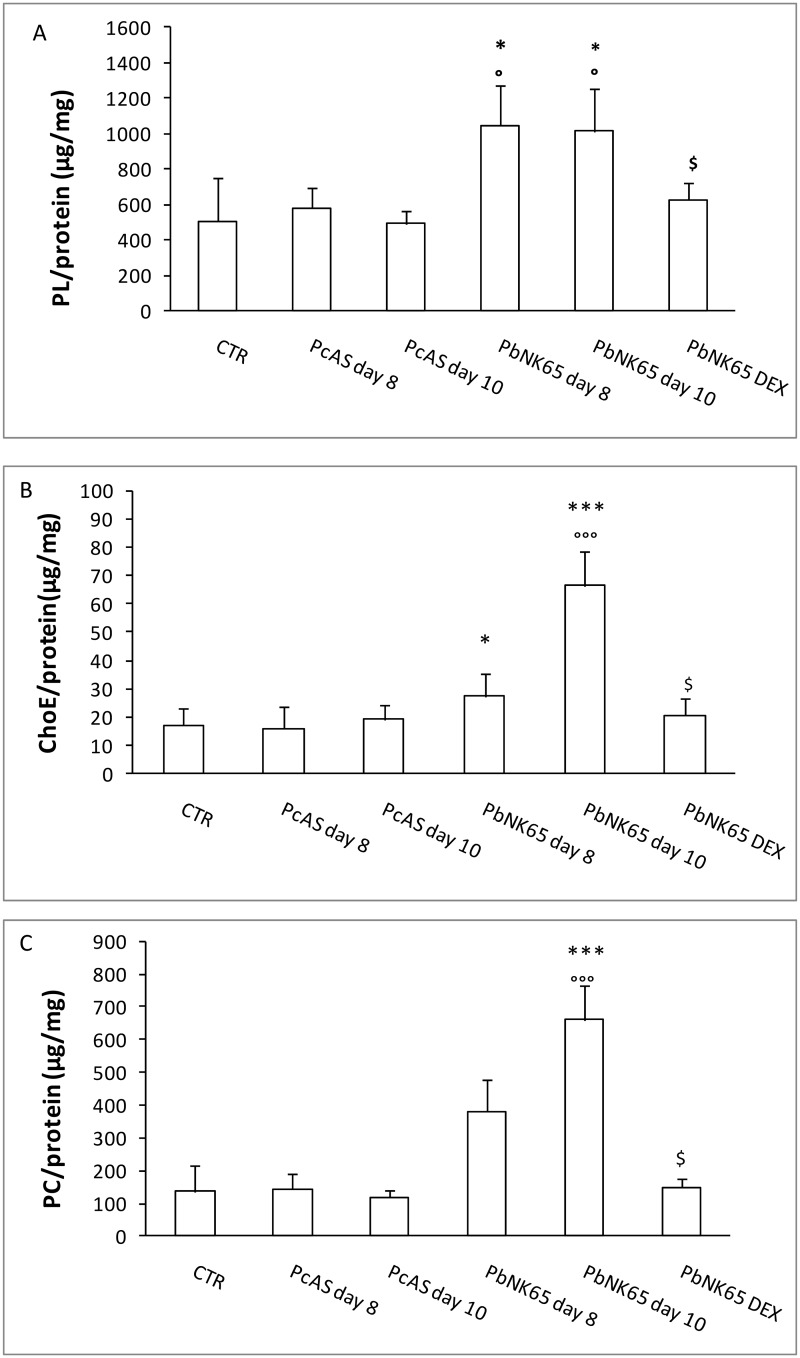
Alteration in the lipid profiles of the lungs from uninfected, *Pb*NK65 or *Pc*AS infected mice. Mice infected with *Pb*NK65 (treated or not intraperitoneally with DEX 80mg/kg) or *Pc*AS were perfused and dissected at day 8 and 10 post infection. The total content of PL (panel A), ChoE (panel B), PC (panel C) /mg protein were determined as described in Materials and Methods. n = 6 mice for each condition; *p<0.05; **p<0.01; ***p<0.0001 vs CTR; °p< 0.05; °°p<0.01; °°°p<0.0001 vs *Pc*AS for each time point; $ p<0.05 *Pb*NK65-DEX vs *Pb*NK65 day 10. PL = phospholipids; ChoE = Cholesterol Esters; PC = phosphatidylcholine.

The lipids of membrane-rich fractions from lung homogenates were analyzed. No differences were found between infected and non-infected control (CTR) mice in the level of free cholesterol (μg Cho/mg protein: CTR 143.5 ± 46¸ *Pc*AS day 10: 124.2 ± 61; *Pb*NK65 day 10: 132.6 ± 40). Significantly higher levels of PLs and esterified Cho (ChoE) were found in lungs obtained from *Pb*NK65-infected mice compared to CTR or *Pc*AS-infected mice at day 8 and 10 post infection (Fig 2A and 2B). Phosphatidylcholine (PC), the most abundant PL found in the lungs, increased in parallel with the increased disease severity (Fig 2C). The treatment with DEX prevented the rise in PLs, ChoE and PC in the lung tissue (Fig 2A, 2B and 2C) at day 10 post infection. No differences were found for phosphatidylethanolamine (PE), sphingomyelin (SM) and phosphatidylinositol (PI) between infected and non-infected CTR mice (not shown).

From the analysis of the fatty acid (FA) profile, the total lung membrane-enriched fraction from *Pb*NK65-infected mice was characterized by higher percentages of palmitic acid (C16:0) and lower percentages of stearic (C18:0) and oleic (C18:1) acid compared to *Pc*AS and CTR mice at both day 8 and 10 post infection (Table 1). The levels of linoleic (C18:2 n-6) acid were also slightly reduced The percentage of docosahexaenoic acid (DHA, C22:6 n-3) was increased by the infection, with no differences between the two parasite strains. The percentages of the other FAs in the total membrane fraction were not affected in the infected animals compared to controls. Due to the high content of DHA, the Peroxidability Index (PI) of the lung tissue from infected mice of both strains was significantly higher compared to CTR mice, indicating a higher relative oxidability of the membranes. The treatment of *Pb*NK65-infected mice with DEX did not significantly affect the fatty acid distribution of lung tissue and consequently the relative oxidability index (PI). In particular, no effect of DEX was found on the levels of DHA (Table 1).

**Table 1:** Fatty acid distribution in the membrane rich fraction of lung tissue from control, *Pc*AS and *Pb*NK65 (treated or not with dexamethasone) infected mice at different times after the infection.

Fatty acids	% of total					
	CTR	*Pc*AS day 8	*Pc*AS day 10	*Pb*NK65 day 8	*Pb*NK65 day 10	*Pb*NK-DEX day 10
**C16:0** (palmitic acid)	28.7 ± 3.0	32.25 ± 8.1	30.2 ± 5.1	35.6 ± 4.0 *	36.7 ± 6.1 *	32.99 ± 8.8
**C16:1** (palmitoleic acid)	2.98 ± 0.1	1.42 ± 1.0	1.21 ± 0.9	3.29 ± 1.2	2.28 ± 1.4	2.95 ± 1.3
**C18:0** (stearic acid)	11.65 ± 2.0	14.01 ± 1.4	13.01 ± 0.4	9.9 ± 1.6 ** ^,^ °°	8.9 ± 1.1 ** ^,^ °°	11.20 ± 1.2 ° ^,^ ^$^
**C18:1** (oleic acid)	15.16 ± 1.2	12.90 ± 0.4	11.98 ± 0.4	9.98 ± 1.1 *** ^,^ °	9.5 ± 0.9 *** ^,^ °	11.22 ± 1.1 * ^,^ ^$$^
**C18:2 n-6** (linoleic acid)	11.71 ± 1.1	11.73 ± 1.0	11.65 ± 1.0	10.5 ± 1.4 * ^,^ °	10.01 ± 1.1 * ^,^ °	11.66 ± 1.2
**C18:3 α** (α-linolenic acid)	1.08 ± 0.9	1.02 ± 0.1	1.0 ± 0.1	1.05 ± 1.6	1.0 ± 0.9	1.25 ± 0.1
**C18:3 γ** (γ-linolenic acid)	2.01 ± 1.1	1.98 ± 0.1	2.04 ± 0.1	1.45 ± 1.2	1.38 ± 1.7	1.98 ± 0.1
**C20:3 n-6** (eicosatrienoic acid)	1.47 ± 0.4	1.74 ± 0.5	1.24 ± 0.5	1.35 ± 0.2	1.75 ± 0.2	1.60 ± 0.3
**C20:4 n-6** (arachidonic acid)	11.67 ± 1.9	12.65 ± 2.5	11.98 ± 2.0	12.01 ± 1.9	12.3 ± 1.01	13.97 ± 2.8
**C20:5 n-3** (eicosapentaenoic acid)	0.40 ± 0.1	0.39 ± 0.2	0.6 ± 0.2	0.42 ± 0.1	0.39 ± 0.1	0.38 ± 0.2
**C22:5 n-3** (docosapentaenoic acid)	2.12 ± 0.2	2.16 ± 0.6	1.99 ± 0.8	1.2 ± 0.2	1.9 ± 0.2	2.06 ± 0.8
**C22:6 n-3** (docosahexanoic acid)	4.07 ± 0.9	6.98 ± 0.4 **	7.1 ± 0.1 **	7.2 ± 0.9 **	8.4 ± 0.8 ***	7.72 ± 0.9 **
**PI** ∞ (Peroxidability index)	111.62	139.35 *	136.62 *	130.99 *	145.94 **	148.51**

*p<0.05;

**p<0.01;

***p<0.001 vs CTR;

°p<0.05;

°°p<0.01 *Pb*NK65 vs *Pc*AS;

^$^ p<0.05;

^$$^p<0.01 *Pb*NK65-DEX vs *Pb*NK65 for each time point.

n = 5–6

^∞^ PI: of lipids. The value is calculated based on the relative oxidation rate of unsatured fatty acids as follows:

PI = (%monoenoic x 0.025)+(%dienoic x 1)+(%trienoic x 3)+(%tetraenoic x 4)+ (%pentaenoic x 6)+(hexaenoic x 8)

By examining the FA profile of PC and ChoE, the two molecular species significantly increased in MA-ARDS, it was found that PC was extremely rich in palmitic acid (48%) and similar in CTR and infected mice, whereas the ChoE fraction in lung membranes of *Pb*NK65-infected mice was characterized by higher percentages of DHA (6.9% vs 3.8% of CTR), linoleic (25.4% vs 16.2% of CTR), and arachidonic acid (26.4% vs 13.7% of CTR) and a higher linoleic/oleic acid ratio (2.3 vs 1.0 of CTR).

### Oxidative damage and fibrosis

In spite of the higher amounts of total membrane PLs present in the lungs of mice with MA-ARDS, and the higher total amounts of polyunsaturated fatty acids (PUFAs), the levels of lipoperoxidation products (TBARS) did not indicate higher oxidative damage in the lungs of infected *Pb*NK65 mice compared to controls or to *Pc*AS-infected mice. In agreement, any fibrotic degeneration was excluded since the levels of hydroxyproline, a marker of collagen deposition, were within the normal range (S1 Fig).

### Lipid changes in plasma

**Fig 3:**
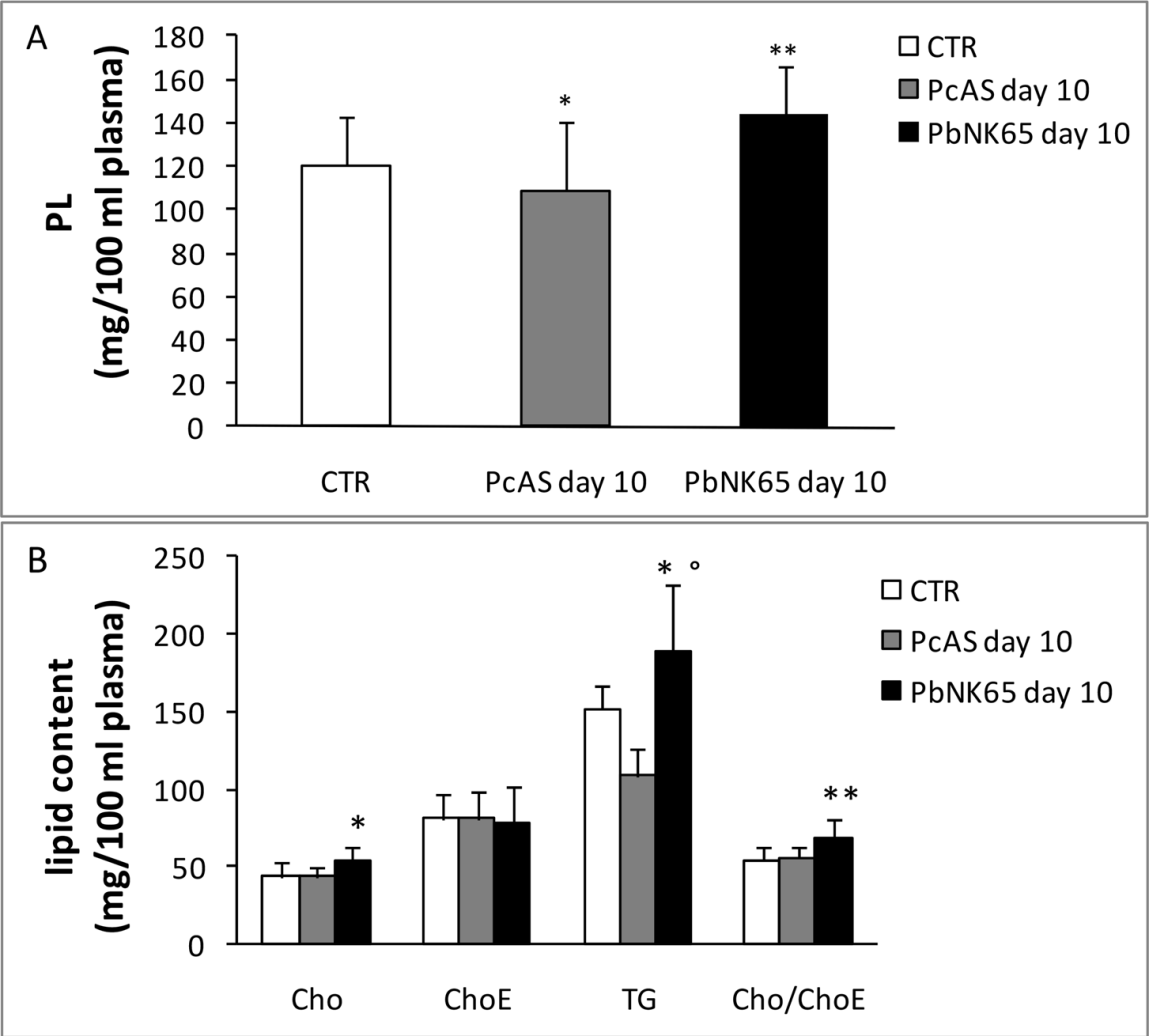
Alteration in the lipid profile of plasma from uninfected, *Pb*NK65 or *Pc*AS infected mice. PL (panel A) and neutral lipid content (panel B) of plasma of infected and uninfected mice at day 10 post infection were determinated as described in Materials and Methods.Cho = Cholesterol, ChoE = Cholesterol esters, TG = Triacylglycerols. *p<0.05; **p<0.01 vs CTR; °p<0.01 *Pb*NK65 vs *Pc*AS. n = 8–12.

**Fig 4:**
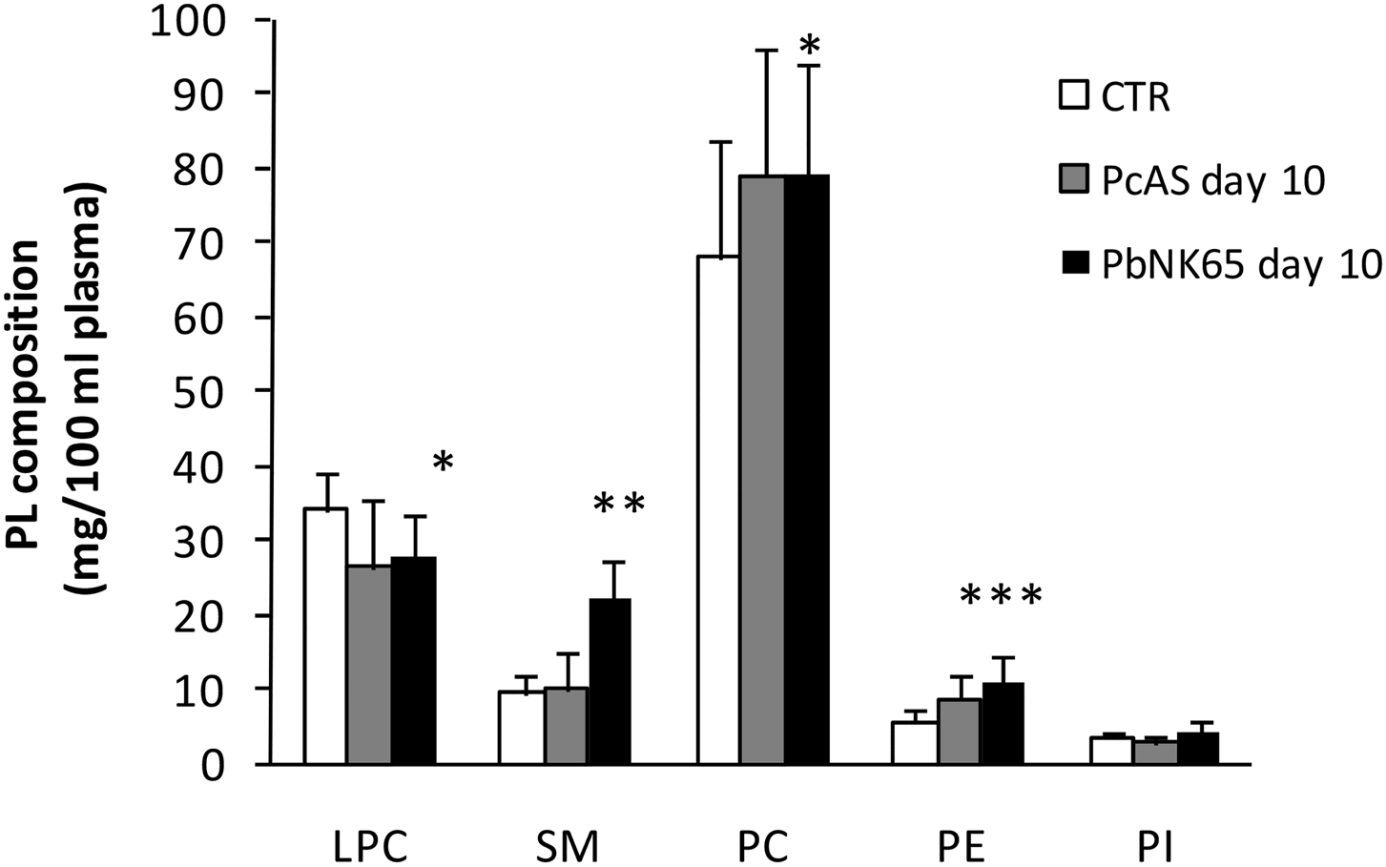
Alteration of the PL composition in plasma from uninfected, *Pb*NK65 or *Pc*AS infected mice. The distribution of plasma PL (mg/100 ml) was determined in uninfected and in *Pb*NK65 or *Pc*AS infected mice at day 10 post infection. LPC = lysophosphatidylcholine; SM = sphingomyelin; PC = phosphatidylcholine; PI = phosphatidylinositol; PE = phosphatidylethanolamine. * p<0.05; ** p<0.01 vs CTR; *** p<0.001 vs CTR. n = 8–12.

Since vascular leakage is a feature of MA-ARDS, and plasma contains a large amount of lipids transported by lipoproteins, it was investigated whether the lipid and FA alterations found in the lungs of *Pb*NK65-infected mice might be related to plasma leakage and lipoprotein infiltration. Compared to CTR or *Pc*AS-infected mice, the lipid profile in plasma of *Pb*NK65-infected mice was significantly modified only at day 10 post infection showing an increase of TG, free Cho and the Cho/ChoE ratio (Fig 3), an increase of PC, PE and SM and a decrease of LPC (Fig 4). Analogously to the lungs, the total fatty acid profile of plasma from *Pb*NK65-infected mice (day 10 post infection) showed lower levels of linoleic acid (C18:2) and higher levels of arachidonic (C20:4) and DHA (C22:6), but similar levels of the saturated palmitic (C16:0) and stearic (C18:0) fatty acids (Table 2). Interestingly, significantly lower percentages of linoleic (27.05 ± 1.1 *Pb*NK65 vs 40.13 ± 2.5 CTR) and higher percentages of arachidonic acid (42.46 ± 0.4 *Pb*NK65 vs 29.34 ± 3.4 CTR) and DHA (8.56 ± 1.6 *Pb*NK65 vs 5.09 ± 0.4 CTR) were also found in the purified plasma (ChoE) fraction.

**Table 2:** Plasma fatty acid distribution (%) at day 10 post infection.

Fatty acids	% of total		
	CTR	*Pc*AS day 10	*Pb*NK65 day 10
**C16:0** (palmitic acid)	19.06 ± 2.8	21.71 ± 1.8	18.11 ± 2.2
**C16:1** (palmitoleic acid)	1.90 ± 1.0	1.27 ± 0.4	0.72 ± 1.1 *
**C18:0** (stearic acid)	11.75 ± 3.5	10.54 ± 2.3	11.48 ± 2.0
**C18:1** (oleic acid)	13.63 ± 1.9	12.98 ± 1.7	13.10 ± 3.7
**C18:2 n-6** (linoleic acid)	33.02 ± 4.8	30.43 ± 3.7	26.90 ± 0.2 *
**C18:3 α** (α-linolenic acid)	0,98 ± 0,4	0,95 ± 0,2	0,47 ± 0.2 *
**C18:3 γ** (γ-linolenic acid)	1.14 ± 0.1	1.17 ± 2.5	0.66 ± 0.3 *
**C20:3 n-6** (eicosatrienoic acid)	13.42 ± 2.8	14.13 ± 2.5	16.25 ± 1.9 *
**C20:4 n-6** (arachidonic acid)	0.49 ± 0.1	0.36 ± 0.1	0.43 ± 0.1
**C20:5 n-3** (eicosapentaenoic acid)	0.39 ± 0.02	0.57± 0.1 *	0.59 ± 0.1 **
**C22:5 n-3** (docosapentaenoic acid)	4.21 ± 1.3	6.48 ± 0.2 *	10.87 ± 1.0 ***
**C22:6 n-3** (docosahexanoic acid)	130.29 ± 28.5	177 ± 21.6	187.78 ± 14.2 *
**PI** ∞ (Peroxidability index)	2.70 ± 0.9	2.18 ± 0.5	1.68 ± 0.4 *

^∞^ PI: Peroxidability index of lipids. The value is calculated based on the relative oxidation rate of unsatured fatty acids as follows:

PI = (%monoenoic x 0.025)+(%dienoic x 1)+(%trienoic x 3)+(%tetraenoic x 4)+ +(%pentaenoic x 6)+(hexaenoic x 8)

*p<0.05;

**p<0.01;

*** p<0.0001 vs CTR

n = 5

### Alteration of the alveolar surfactant composition in murine MA-ARDS

**Fig 5:**
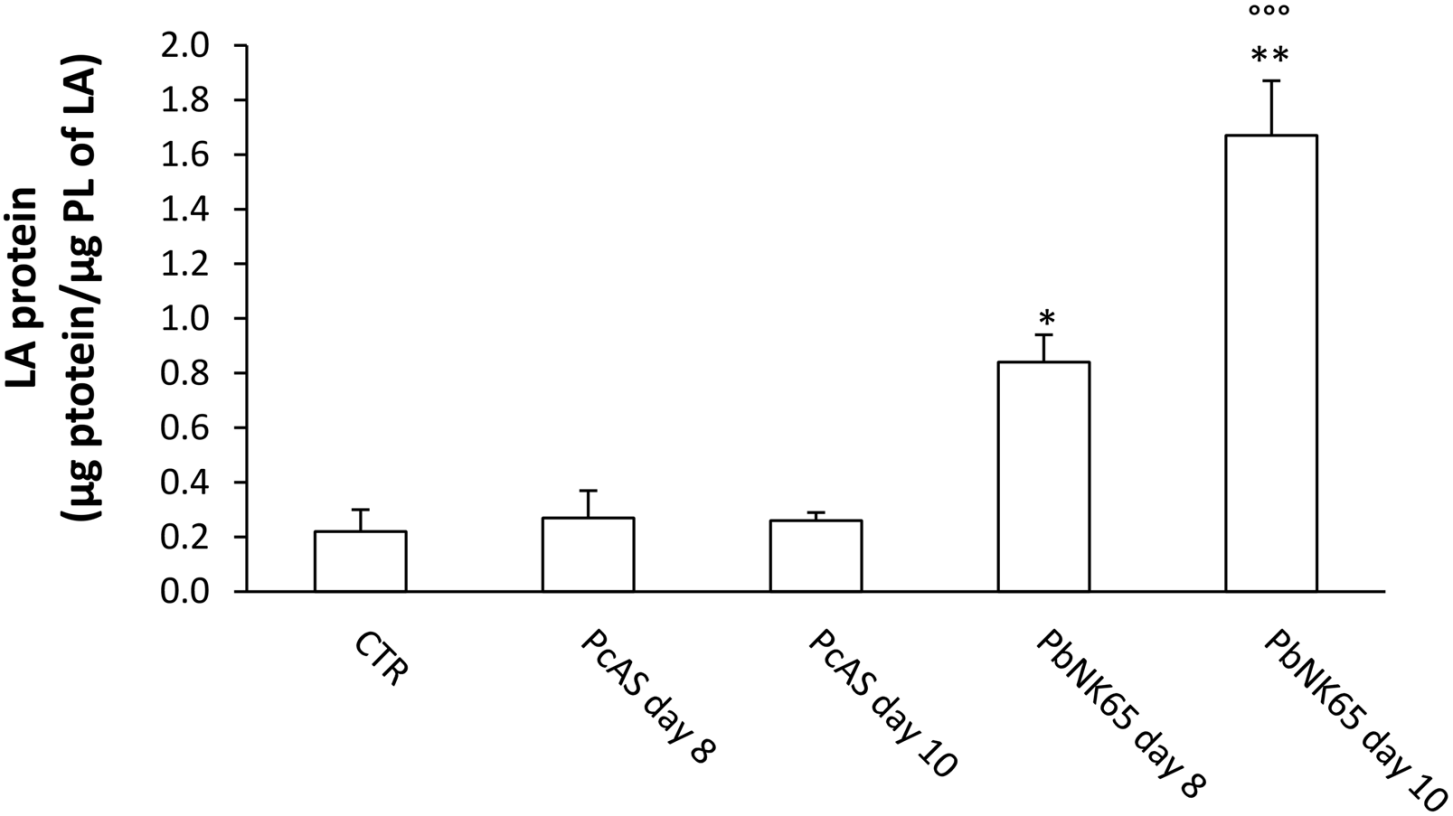
Protein content of the Large Aggregate (LA) fraction of BAL fluid from uninfected, *Pb*NK65 or *Pc*AS infected mice. Bronchoalveolar lavage (BAL) fluid samples were collected from control mice and mice infected with *Pb*NK65 (n = 10 for day 8 post infection, n = 13 for day 10 post infection), or *Pc*AS (n = 8 for day 8 or 10 post infection). The large aggregate fraction (LA) was isolated and the content of proteins was determined. *p<0.05; **p<0.01; ***p<0.001 vs CTR; °°°p<0.001 vs *Pc*AS for each time point.

**Fig 6:**
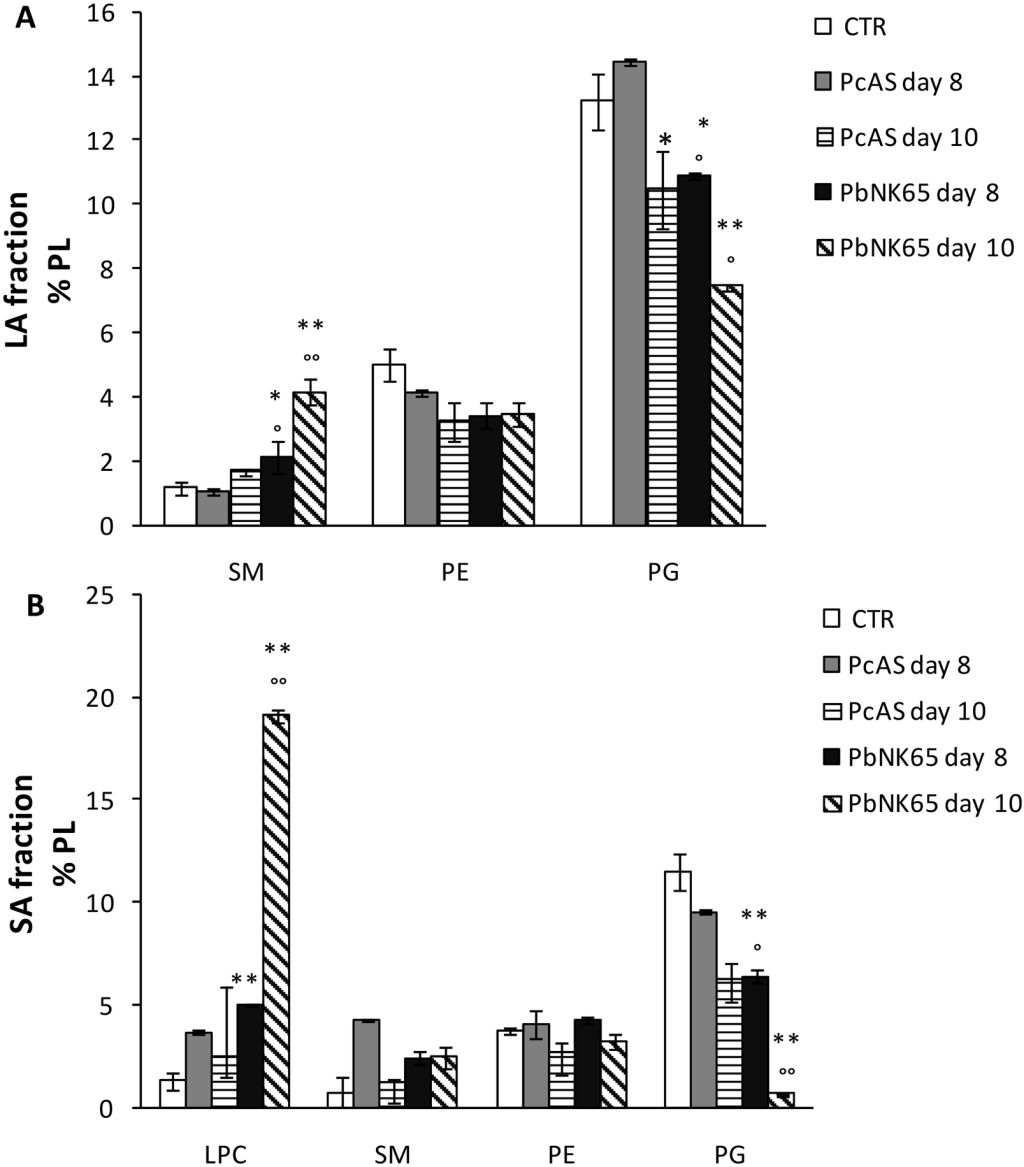
Alterations in the lipid profile of the pulmonary surfactant of *Pb*NK65 or *Pc*AS infected mice. (Panel A) Large aggregate (LA) and (Panel B) small aggregate (SA) fractions of pulmonary surfactant were isolated from the BAL fluid of control mice and mice infected with *Pb*NK65 or *Pc*AS and the percentage distribution of PL was determined. SM = Sphingomyelin, PE = phosphatidylethanolamine, PG = phosphatidylglycerol and LPC = lysophosphatidylcholine. n = 5–7; * p< 0.05 vs CTR; ** p<0.001 vs CTR; ° p< 0.05, °° p< 0.01 vs *Pc*AS for each time point.

The total (cell-free) BAL fluid of *Pc*AS-infected mice showed a limited, although significant, increase in protein levels at 8 and 10 days post infection (Table 3). The increase in protein content was more pronounced in mice infected with *Pb*NK65 at each time point analyzed. PLs were also significantly increased in BAL in both *Pc*AS and *Pb*NK65-infected mice, with the highest levels detected at day 10 in *Pb*NK65-infected mice (Table 3). The amount of the LA fraction obtained from *Pb*NK65- or *Pc*AS-infected mice was not significantly different from that of non-infected CTR mice (S2 Fig). However, the composition of the LA fraction was significantly altered in *Pb*NK65-infected mice at day 10 post infection, being characterized by an increased protein content (Fig 5) and a modified PL composition (Fig 6A). A significant increase in the relative amounts of SM and a decrease in phosphatidylglycerol (PG) were observed, whereas the percentages of PC and PE were not altered (Fig 6A and data not shown). The PL composition of the SA fraction was altered as well, with almost undetectable PG and a dramatic increase in LPC, the hydrolysis product of PC (Fig 6B).

**Table 3:** Total protein and phospholipid (PL) content in BAL fluid of *Pc*AS or *Pb*NK65 infected mice at different time points after the infection.

	CTR	*Pc*AS day 8	*Pc*AS day 10	*Pb*NK65 day 8	*Pb*NK65 day 10
**Protein (μg/ml)**	61.41 ± 16.3	97.57 ± 22.7 *	92.18 ± 13.0 *	635.52 ± 352.3 *; °	1630.27 ± 142.1 **;°°
**PL (μg/ml)**	36.32 ± 3.4	91.76 ± 32.0*	80.12 ± 30.6*	77.63 ± 33.8*	162.3 ± 75.8**
**Protein/PL**	1.71 ± 0.5	1.06 ± 1.0	1.15 ± 0.6	8.18 ± 8.0 **	10.05 ± 5.5 **

*p<0.05;

**p<0.0001 vs CTR;

° p<0.05;

°°p<0.001 vs *Pc*AS n = 3–4, n = 5

## Discussion

This study illustrates that the lipid and FA profile of lung tissue is significantly altered during murine MA-ARDS. Increased levels of PLs and ChoE were found in the membrane-enriched fraction of *Pb*NK65-infected mice, which develop significant lung pathology, compared to *Pc*AS-infected mice or uninfected controls. *Pb*NK65-infected mice also manifest higher parasitaemia and higher hemozoin deposition than *Pc*AS-infected mice. The increase in lung PL is a common, although not universal response to pulmonary inflammation and may be partially explained by an increase of intra or extracellular surfactant [37, 38]. In fact, PC, the main PL of surfactant, was also increased in the lung tissue of *Pb*NK65-infected mice and was particularly rich in palmitic acid, the most abundant fatty acid of surfactant [39]. However, the increased levels of palmitic acid may be also partially contributed by an impairment of the elongation pathway to stearic (C18:0) and desaturation to oleic acid (C18:1).

The increase of ChoE suggests a correlation with interstitial oedema and lipoprotein infiltration occurring during MA-ARDS. Indeed, plasma is rich in ChoE-containing lipoproteins, which may leak out of the vessels upon increased permeability. This is confirmed by the observation that in *Pc*AS mice, which show no signs of oedema, the levels of ChoE are low. The presence of oedema, inflammatory cells and infected erythrocytes is an hallmark of both human and experimental MA-ARDS caused by different Plasmodium species [9]. The lipoprotein infiltration is suggested also by the fatty acid composition of ChoE from *Pb*NK65-infected lungs, which reflects the peculiar fatty acid composition of plasma ChoE: high linoleic, arachidonic and DHA fatty acids and high linoleic/oleic fatty acids ratio. The high linoleic/oleic ratio of plasma ChoE indicates that the majority of the ChoE is derived from the activity of the plasma Lecithin Cholesterol AcylCoA Transferase (LCAT), shown to be highly specific for linoleic acid, rather than from the hepatic AcylCoA Cholesterol Acyl Transferase (ACAT) activity, more specific for oleic acid [40]. Differently from the increase of PLs and ChoE, the increase of DHA seems to be related to the malaria infection and not to the lung pathology since it is present also in *Pc*AS-infected mice and is not inhibited by DEX treatment. Moreover, we tend to exclude that the modification of plasma lipid content and composition are related to the lysis of infected erythrocytes. In fact, preliminary data indicate that there are no differences in the PL composition of RBC plasma membranes from *Pb*NK65- infected mice compared to control or *Pc*AS- infected mice. Furthermore, the high levels of TG present in the plasma cannot be attributed to RBS lysis since TG are found in trace amounts in RBC membranes. High levels of TG as well as PE may derive from an impairment of lipoprotein lipase (LPL) activity of *Pb*NK65-infected mice, as reported in other models of hyperlipidemia related to infection and inflammation [41, 42]. Based on these observations, the changes in plasma lipid composition may be related to the heavy inflammatory response present in the lungs of *Pb*NK65-infected mice which leads to altered surfactant properties and increased lung pathology.

Previously, we have shown that hemozoin contributes to pulmonary inflammation during murine MA-ARDS [15]. In addition to its inflammatory effects, hemozoin also has a strong pro-oxidant activity [43]. In this study, lung hemozoin levels were higher in *Pb*NK65-infected mice with pulmonary pathology compared to *Pc*AS-infected mice with no signs of pulmonary pathology. However, in spite of the relatively high hemozoin concentration and the increased amounts of PUFAs present in the membranes of *Pb*NK65-infected mice, no sign of increased lipid peroxidation was detected in the lungs in the present study. The lung is an organ that—due to its role in breathing—is very much exposed to oxygen and thus to oxidative insults. Therefore, it has developed strong antioxidative defense mechanisms [44]. It is thus possible that the oxidative stress and lipoperoxidation occuring in the lungs of mice with MA-ARDS, do not exceed the antioxidant defenses of the lung tissue, thus significant oxidative damage is not observed. Consistently with the absence of oxidative damage, pulmonary fibrosis was also absent in the lungs of the studied mice. The latter may also be related to the acute nature of MA-ARDS, as fibro-proliferation mainly develops during the subsequent, more chronic phase of ARDS. Similar observations are reported from the post-mortem analysis of patients with MA-ARDS, who usually do not demonstrate fibrosis unless they die after a long chronic period of MA-ARDS [45, 46]

The surfactant is synthesized by type II pneumocytes in the epithelial lining of the alveoli and secreted into the alveolar space. Increased surfactant levels may be due to an augmented synthesis and/or impaired endocytosis of surfactant by type II cells, which might occur when type II cell membranes are damaged or when plasma proteins are present in the alveolar space. Pulmonary surfactant has a crucial role in the normal physiology of the lungs [47], and our data suggest that the intrinsic activity of pulmonary surfactant may be significantly decreased during murine MA-ALI/ARDS [47, 48]. In fact in the *Pb*NK65-infected mice, vascular leakage and pulmonary oedema result in increased protein levels, either in the total BAL supernatant or in the BAL LA fraction. Plasma proteins are known to decrease the intrinsic surface activity of surfactant [49]. Furthermore, an altered PL profile was detected in the LA fraction, which was characterized by higher levels of SM and lower levels of PG. PG was also dramatically decreased in the SA fraction to almost undetectable levels. PG is normally squeezed out of the surfactant layer during compression and has been suggested to aid in the preferential enrichment of DPPC in the surfactant film [39]. Similar changes in the PL composition have been reported in other animal models of lung injury and in patients with established ARDS [25, 50] and may be related to the altered surfactant reuptake, secretion, and/or synthesis by injured alveolar cells or to PL contamination due to inflammatory cells. The increase of LPC in the SA fraction is consistent with the action of secretory PLA_2_, whose activity has been detected in BAL fluid of ARDS patients, in particular in the SA aggregates [51]. LPC is a known inhibitor of surfactant activity and may further worsen lung functions [52].

## Conclusion

Experimental MA-ARDS is associated with severe inflammation, oedema and, as shown here, modifications of the lipid profile of both lung tissue and pulmonary surfactant. Total PLs, and PC in particular, together with ChoE levels were significantly increased in lung tissue. The fatty acid distribution was also altered during lung pathology, with increased palmitic acid and decreased stearic and oleic acid levels. With regard to the alveolar surfactant, a significant increase in the protein content was found, probably due to vascular leakage. Alterations in the PL composition, with increased levels of LPC and SM, and decreased levels of PG were also observed. Since LPC is a known inhibitor of surfactant activity, these changes together with the intense inflammation, may contribute to the impairment of lung functions and to the severity of murine MA-ALI/ARDS.

## Supporting Information

S1 Fig Oxidative damage and fibrosis in lung tissue of *Pb*NK65 or *Pc*AS infected mice. MDA content (A) (pmole/mg protein) and OH-proline content (μg/mg protein) (B) in infected and non infected mice at day 8 or 10 days post infection. n = 7–8.
(TIF)

S2 Fig Amount of the LA fraction of *Pb*NK65 or *Pc*AS infected mice. Percentage of LA fraction obtained from uninfected or *Pb*NK65- or *Pc*AS-infected mice at 8 or 10 days post infection.
(TIF)

## References

[1] McGready R, Wongsaen K, Chu CS, Tun NW, Chotivanich K, White NJ, et al Uncomplicated Plasmodium vivax malaria in pregnancy associated with mortality from acute respiratory distress syndrome. Malar J. 2014;13:191 10.1186/1475-2875-13-191 24886559 PMC4046059

[2] WHO. Guidelines for the treatment of malaria. World Health Organization. 2010;2nd Ed, Geneva. 25473692

[3] Ampawong S, Chaisri U, Viriyavejakul P, Prapansilp P, Grau GE, Turner GD, et al A potential role for interleukin-33 and γ-epithelium sodium channel in the pathogenesis of human malaria associated lung injury. Malar J. 2015;14(1):389 10.1186/s12936-015-0922-x 26437894 PMC4595310

[4] Gupta D, Ramanathan RP, Aggarwal AN, Jindal SK. Assessment of factors predicting outcome of acute respiratory distress syndrome in North India. Respirology (Carlton, Vic. 2001;6(2):125–30. . 11422891 10.1046/j.1440-1843.2001.00324.x

[5] Taylor WR, Hanson J, Turner GD, White NJ, Dondorp AM. Respiratory manifestations of malaria. Chest. 2012;142(2):492–505. 10.1378/chest.11-2655 . 22871759

[6] William T, Menon J, Rajahram G, Chan L, Ma G, Donaldson S, et al Severe Plasmodium knowlesi malaria in a tertiary care hospital, Sabah, Malaysia. Emerg Infect Dis. 2011;17(7):1248–55. 10.3201/eid1707.101017 21762579 PMC3381373

[7] Van den Steen PE, Geurts N, Deroost K, Van Aelst I, Verhenne S, Heremans H, et al Immunopathology and dexamethasone therapy in a new model for malaria-associated acute respiratory distress syndrome. Am J Respir Crit Care Med. 2010;181(9):957–68. 10.1164/rccm.200905-0786OC . 20093644

[8] Epiphanio S, Campos MG, Pamplona A, Carapau D, Pena AC, Ataíde R, et al VEGF promotes malaria-associated acute lung injury in mice. PLoS Pathog. 2010;6(5):e1000916 10.1371/journal.ppat.1000916 20502682 PMC2873913

[9] Aitken EH, Negri EM, Barboza R, Lima MR, Álvarez JM, Marinho CR, et al Ultrastructure of the lung in a murine model of malaria-associated acute lung injury/acute respiratory distress syndrome. Malar J. 2014;13:230 10.1186/1475-2875-13-230 24927627 PMC4062769

[10] Craig AG, Grau GE, Janse C, Kazura JW, Milner D, Barnwell JW, et al The role of animal models for research on severe malaria. PLoS Pathog. 2012;8(2):e1002401 10.1371/journal.ppat.1002401 22319438 PMC3271056

[11] Mohan A, Singh P, Kumar S, Mohan C, Pathak AK, Pandey RM, et al Effect of change in symptoms, respiratory status, nutritional profile and quality of life on response to treatment for advanced non-small cell lung cancer. Asian Pac J Cancer Prev. 2008;9(4):557–62. . 19256738

[12] Van den Steen PE, Deroost K, Deckers J, Van Herck E, Struyf S, Opdenakker G. Pathogenesis of malaria-associated acute respiratory distress syndrome. Trends Parasitol. 2013;29(7):346–58. 10.1016/j.pt.2013.04.006 . 23742967

[13] Milner D, Factor R, Whitten R, Carr RA, Kamiza S, Pinkus G, et al Pulmonary pathology in pediatric cerebral malaria. Hum Pathol. 2013;44(12):2719–26. 10.1016/j.humpath.2013.07.018 24074535 PMC3838443

[14] Lacerda MV, Fragoso SC, Alecrim MG, Alexandre MA, Magalhães BM, Siqueira AM, et al Postmortem characterization of patients with clinical diagnosis of Plasmodium vivax malaria: to what extent does this parasite kill? Clin Infect Dis. 2012;55(8):e67–74. 10.1093/cid/cis615 . 22772803

[15] Deroost K, Tyberghein A, Lays N, Noppen S, Schwarzer E, Vanstreels E, et al Hemozoin Induces Lung Inflammation and Correlates with Malaria-Associated Acute Respiratory Distress Syndrome. Am J Respir Cell Mol Biol. . 23328641 10.1165/rcmb.2012-0450OC

[16] Esterbauer H, Schaur RJ, Zollner H. Chemistry and biochemistry of 4-hydroxynonenal, malonaldehyde and related aldehydes. Free Radic Biol Med. 1991;11(1):81–128. . 1937131 10.1016/0891-5849(91)90192-6

[17] Niki E. Lipid peroxidation: physiological levels and dual biological effects. Free Radic Biol Med. 2009;47(5):469–84. 10.1016/j.freeradbiomed.2009.05.032 . 19500666

[18] Bursten SL, Federighi DA, Parsons P, Harris WE, Abraham E, Moore EE, et al An increase in serum C18 unsaturated free fatty acids as a predictor of the development of acute respiratory distress syndrome. Crit Care Med. 1996;24(7):1129–36. . 8674324 10.1097/00003246-199607000-00011

[19] Quinlan GJ, Lamb NJ, Evans TW, Gutteridge JM. Plasma fatty acid changes and increased lipid peroxidation in patients with adult respiratory distress syndrome. Crit Care Med. 1996;24(2):241–6. . 8605795 10.1097/00003246-199602000-00010

[20] Lopez-Rodriguez E, Pérez-Gil J. Structure-function relationships in pulmonary surfactant membranes: from biophysics to therapy. Biochim Biophys Acta. 2014;1838(6):1568–85. 10.1016/j.bbamem.2014.01.028 . 24525076

[21] Ochiai R. Mechanical ventilation of acute respiratory distress syndrome. J Intensive Care. 2015;3(1):25 10.1186/s40560-015-0091-6 26045965 PMC4456061

[22] Van Golde LM, Den Breejen JN, Batenburg JJ. Isolated alveolar type II cells: a model for studies on the formation of surfactant dipalmitoylphosphatidylcholine. Biochem Soc Trans. 1985;13(6):1087–9. . 3841521 10.1042/bst0131087

[23] Parra E, Pérez-Gil J. Composition, structure and mechanical properties define performance of pulmonary surfactant membranes and films. Chem Phys Lipids. 2015;185C:153–75. 10.1016/j.chemphyslip.2014.09.002 . 25260665

[24] Günther A, Ruppert C, Schmidt R, Markart P, Grimminger F, Walmrath D, et al Surfactant alteration and replacement in acute respiratory distress syndrome. Respir Res. 2001;2(6):353–64. 11737935 10.1186/rr86 PMC64803

[25] Gunther A, Schmidt R, Harodt J, Schmehl T, Walmrath D, Ruppert C, et al Bronchoscopic administration of bovine natural surfactant in ARDS and septic shock: impact on biophysical and biochemical surfactant properties. Eur Respir J. 2002;19(5):797–804. . 12030716 10.1183/09031936.02.00243302

[26] Raghavendran K, Davidson BA, Knight PR, Wang Z, Helinski J, Chess PR, et al Surfactant dysfunction in lung contusion with and without superimposed gastric aspiration in a rat model. Shock. 2008;30(5):508–17. 10.1097/SHK.0b013e3181673fc5 18323743 PMC2692208

[27] Holm BA, Wang Z, Notter RH. Multiple mechanisms of lung surfactant inhibition. Pediatr Res. 1999;46(1):85–93. . 10400140 10.1203/00006450-199907000-00015

[28] Lema G, Enhorning G. Surface properties after a simulated PLA2 hydrolysis of pulmonary surfactant's main component, DPPC. Biochim Biophys Acta. 1997;1345(1):86–92. . 9084505 10.1016/s0005-2760(96)00180-4

[29] Davidson BA, Knight PR, Wang Z, Chess PR, Holm BA, Russo TA, et al Surfactant alterations in acute inflammatory lung injury from aspiration of acid and gastric particulates. American journal of physiology. 2005;288(4):L699–708. . 15757954 10.1152/ajplung.00229.2004

[30] Deroost K, Lays N, Noppen S, Martens E, Opdenakker G, Van den Steen PE. Improved methods for haemozoin quantification in tissues yield organ-and parasite-specific information in malaria-infected mice. Malar J. 11:166 10.1186/1475-2875-11-166 22583751 PMC3473299

[31] Folch J, Lees M, Sloane Stanley GH. A simple method for the isolation and purification of total lipides from animal tissues. J Biol Chem. 1957;226(1):497–509. . 13428781

[32] Bartlett GR. Phosphorus assay in column chromatography. J Biol Chem. 1959;234(3):466–8. . 13641241

[33] Svennerholm L. The quantitative estimation of cerebrosides in nervous tissue. J Neurochem. 1956;1(1):42–53. . 13346373 10.1111/j.1471-4159.1956.tb12053.x

[34] Corsetto PA, Montorfano G, Zava S, Jovenitti IE, Cremona A, Berra B, et al Effects of n-3 PUFAs on breast cancer cells through their incorporation in plasma membrane. Lipids Health Dis. 2011;10:73 10.1186/1476-511X-10-73 21569413 PMC3127786

[35] D'Alessandro S, Basilico N, Corbett Y, Scaccabarozzi D, Omodeo-Sale F, Saresella M, et al Hypoxia modulates the effect of dihydroartemisinin on endothelial cells. Biochem Pharmacol. 82(5):476–84. 10.1016/j.bcp.2011.06.002 21684264

[36] Tager AM, Kradin RL, LaCamera P, Bercury SD, Campanella GS, Leary CP, et al Inhibition of pulmonary fibrosis by the chemokine IP-10/CXCL10. Am J Respir Cell Mol Biol. 2004;31(4):395–404. . 15205180 10.1165/rcmb.2004-0175OC

[37] Viviano CJ, Bakewell WE, Dixon D, Dethloff LA, Hook GE. Altered regulation of surfactant phospholipid and protein A during acute pulmonary inflammation. Biochim Biophys Acta. 1995;1259(3):235–44. . 8541330 10.1016/0005-2760(95)00167-0

[38] Tibboel J, Reiss I, de Jongste JC, Post M. Sphingolipids in lung growth and repair. Chest. 2014;145(1):120–8. 10.1378/chest.13-0967 . 24394822

[39] Veldhuizen R, Nag K, Orgeig S, Possmayer F. The role of lipids in pulmonary surfactant. Biochim Biophys Acta. 1998;1408(2–3):90–108. . 9813256 10.1016/s0925-4439(98)00061-1

[40] Hodson L, Skeaff CM, Fielding BA. Fatty acid composition of adipose tissue and blood in humans and its use as a biomarker of dietary intake. Prog Lipid Res. 2008;47(5):348–80. 10.1016/j.plipres.2008.03.003 . 18435934

[41] Memon RA, Holleran WM, Moser AH, Seki T, Uchida Y, Fuller J, et al Endotoxin and cytokines increase hepatic sphingolipid biosynthesis and produce lipoproteins enriched in ceramides and sphingomyelin. Arterioscler Thromb Vasc Biol. 1998;18(8):1257–65. . 9714132 10.1161/01.atv.18.8.1257

[42] Wu G, Brouckaert P, Olivecrona T. Rapid downregulation of adipose tissue lipoprotein lipase activity on food deprivation: evidence that TNF-alpha is involved. Am J Physiol Endocrinol Metab. 2004;286(5):E711–7. . 14693508 10.1152/ajpendo.00257.2003

[43] Schwarzer E, Muller O, Arese P, Siems WG, Grune T. Increased levels of 4-hydroxynonenal in human monocytes fed with malarial pigment hemozoin. A possible clue for hemozoin toxicity. FEBS letters. 1996;388(2–3):119–22. . 8690068 10.1016/0014-5793(96)00523-6

[44] Rahman I, Biswas SK, Kode A. Oxidant and antioxidant balance in the airways and airway diseases. Eur J Pharmacol. 2006;533(1–3):222–39. 10.1016/j.ejphar.2005.12.087 . 16500642

[45] Charoenpan P, Indraprasit S, Kiatboonsri S, Suvachittanont O, Tanomsup S. Pulmonary edema in severe falciparum malaria. Hemodynamic study and clinicophysiologic correlation. Chest. 1990;97(5):1190–7. . 2184996 10.1378/chest.97.5.1190

[46] Feldman RM, Singer C. Noncardiogenic pulmonary edema and pulmonary fibrosis in falciparum malaria. Reviews of infectious diseases. 1987;9(1):134–9. . 3547568 10.1093/clinids/9.1.134

[47] Zhao CZ, Fang XC, Wang D, Tang FD, Wang XD. Involvement of type II pneumocytes in the pathogenesis of chronic obstructive pulmonary disease. Respir Med. 2010;104(10):1391–5. 10.1016/j.rmed.2010.06.018 . 20638828

[48] Müller B, Garn H, Hochscheid R. Impaired recycling of surfactant-like liposomes in type II pneumocytes from injured lungs. Thorax. 2003;58(2):127–34. 12554895 10.1136/thorax.58.2.127 PMC1746577

[49] Holm BA, Enhorning G, Notter RH. A biophysical mechanism by which plasma proteins inhibit lung surfactant activity. Chem Phys Lipids. 1988;49(1–2):49–55. . 3233711 10.1016/0009-3084(88)90063-1

[50] Veldhuizen RA, McCaig LA, Akino T, Lewis JF. Pulmonary surfactant subfractions in patients with the acute respiratory distress syndrome. Am J Respir Crit Care Med. 1995;152(6 Pt 1):1867–71. . 8520748 10.1164/ajrccm.152.6.8520748

[51] Nakos G, Kitsiouli E, Hatzidaki E, Koulouras V, Touqui L, Lekka ME. Phospholipases A2 and platelet-activating-factor acetylhydrolase in patients with acute respiratory distress syndrome. Crit Care Med. 2005;33(4):772–9. . 15818104 10.1097/01.ccm.0000158519.80090.74

[52] Wang Z, Notter RH. Additivity of protein and nonprotein inhibitors of lung surfactant activity. Am J Respir Crit Care Med. 1998;158(1):28–35. . 9655703 10.1164/ajrccm.158.1.9709041

