# Supplementary figures and images for: Altered Lipid Composition of Surfactant and Lung Tissue in Murine Experimental Malaria-Associated Acute Respiratory Distress Syndrome
Source: PLoS One. 2015 Dec 1;10(12):e0143195. doi: 10.1371/journal.pone.0143195 (PMC4666673; doi:10.1371/journal.pone.0143195)

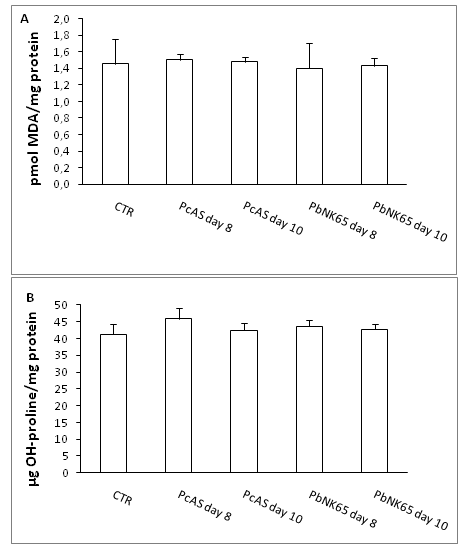

Supplement: S1 Fig — MDA content (A) (pmole/mg protein) and OH-proline content (μg/mg protein) (B) in infected and non infected mice at day 8 or 10 days post infection. n = 7–8. (TIF) [file pone.0143195.s001.tif]

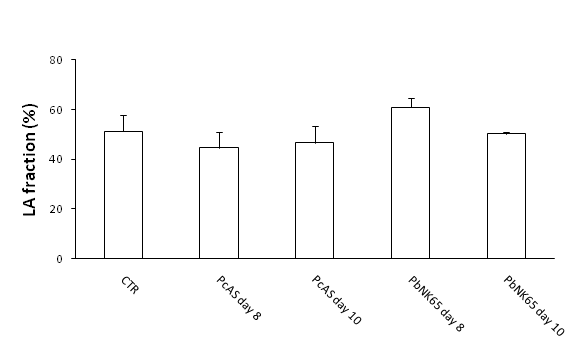

Supplement: S2 Fig — Percentage of LA fraction obtained from uninfected or PbNK65- or PcAS-infected mice at 8 or 10 days post infection. (TIF) [file pone.0143195.s002.tif]
